# The TrxG Complex Mediates Cytokine Induced *De Novo* Enhancer Formation in Islets

**DOI:** 10.1371/journal.pone.0141470

**Published:** 2015-10-27

**Authors:** Bryan R. Tennant, Peter Hurley, Jasmine Dhillon, Amol Gill, Cheryl Whiting, Brad G. Hoffman

**Affiliations:** 1 Child and Family Research Institute, British Columbia Children’s Hospital and Sunny Hill Health Centre, 950 W28th Avenue, Vancouver, British Columbia, Canada; 2 Department of Surgery, University of British Columbia, Vancouver, B.C., Canada; NIDCR/NIH, UNITED STATES

## Abstract

To better understand how β-cells respond to proinflammatory cytokines we mapped the locations of histone 3 lysine 4 monomethylation (H3K4me1), a post-translational histone modification enriched at active and poised cis-regulatory regions, in IFNγ, Il-1β, and TNFα treated pancreatic islets. We identified 96,721 putative cis-regulatory loci, of which 3,590 were generated *de novo*, 3,204 had increased H3K4me1, and 5,354 had decreased H3K4me1 in IFNγ, Il-1β, and TNFα exposed islets. Roughly 10% of the *de novo* and increased regions were enriched for the repressive histone modification histone 3 lysine 27 trimethylation (H3K27me3) in untreated cells, and these were frequently associated with chemokine genes. We show that IFNγ, Il-1β, and TNFα exposure overcomes this repression and induces chemokine gene activation in as little as three hours, and that this expression persists for days in absence of continued IFNγ, Il-1β, and TNFα exposure. We implicate trithorax group (TrxG) complexes as likely players in the conversion of these repressed loci to an active state. To block the activity of these complexes, we suppressed *Wdr5*, a core component of the TrxG complexes, and used the H3K27me3 demethylase inhibitor GSK-J4. We show that GSK-J4 is particularly effective in blunting IFNγ, Il-1β, and TNFα-induced chemokine gene expression in β-cells; however, it induced significant islet-cell apoptosis and β-cell dysfunction. *Wdr5* suppression also reduced IFNγ, Il-1β, and TNFα induced chemokine gene expression in β-cells without affecting islet-cell survival or β-cell function after 48hrs, but did begin to increase islet-cell apoptosis and β-cell dysfunction after four days of treatment. Taken together these data suggest that the TrxG complex is potentially a viable target for preventing cytokine induced chemokine gene expression in β-cells.

## Introduction

Type 1 diabetes mellitus (T1D) is an autoimmune disease in which the insulin-producing β-cells in the pancreas are selectively destroyed by the host immune system [1–3]. The auto-immunological assault on β-cells comprises multiple immune cell types including macrophages and auto-reactive T cells, which act in part by secreting proinflammatory cytokines, including IFNγ, Il-1β, and TNFα [3]. In the early phases of T1D development exposure of β-cells to these cytokines is thought to induce β-cell dysfunction and the expression of chemokines and cytokines, such as Ccl2 (Mcp-1), Ccl20 (Mip-3α), and Cxcl10 (Ip-10) that increase the infiltration of the islets by immune-cells [3–8]. This results in the amplification of the immune response, ultimately leading to β-cell death and diabetes development.

Layered on top of an organism’s DNA sequence are numerous types of heritable epigenetic information, including various types of post-translational histone modifications [9,10]. Many of these histone modifications are altered in response to different cellular stimuli and act as central determinants in mediating subsequent gene expression changes. For example, gains in histone acetylation are typically associated with gene activation [10,11]; whereas, histone methylations such as the mono-, di-, or trimethylation of histone 3 lysine 4 (H3K4me1, H3K4me2, H3K4me3) are associated with gene activation, and the trimethylation of histone 3 lysine 9 (H3K9me3) or 27 (H3K27me3) are associated with gene repression [10,12,13].

Chronic exposure to inflammatory cytokines alters gene expression patterns in many tissues, and epigenetic alterations have been identified as critical mediators of these changes [14–16]. For example, cytokine exposure can induce the removal of repressive marks at regulatory regions for various proinflammatory genes in macrophages [17,18], and in smooth muscle cells in a mouse model of Type 2 diabetes [19]. In β-cells, although it is well established that proinflammatory cytokines cause the activation of chemokine and cytokine genes, the mechanisms behind these changes are not clear. We predicted that cytokine exposure would induce specific epigenetic changes in β-cells, and that these epigenetic changes would be essential for cytokine induced β-cell chemokine and cytokine gene expression. We further hypothesized that treatments designed to block these epigenetic changes may prevent cytokine induced β-cell chemokine and cytokine gene expression. In support of this hypothesis treatment of syngeneic and allogeneic islet graft recipient mice with histone deacetlyase inhibitors (HDACi’s) reduces cytokine induced β-cell dysfunction, thereby improving islet graft survival [20–23]. As such, we believe that drugs targeting chromatin-remodeling factors may promise new treatments for early-diagnosed patients with T1D to help prevent escalation of the β-cell immune assault and prevent loss of β-cell functional maturity.

Given the association of specific histone modifications with different types of regulatory elements, the mapping of various histone modifications using chromatin immunoprecipitation followed by next generation sequencing (ChIP-seq) has become a useful strategy in the identification of regulatory elements genome-wide [24–28]. For example, we previously determined that H3K4me1 is particularly useful in the identification of active and poised cis-regulatory loci, such as promoter and enhancer regions, in islets [29,30]. Thus, to begin to determine what specific chromatin modification changes are induced in β-cells by exposure to proinflammatory cytokines, so as to gain insight into which chromatin-remodeling factors may be useful therapeutic targets, we sought to map the distribution of H3K4me1 in cytokine treated islets.

## Results

### Cytokine exposure induces *de novo* enhancer formation in in insi

**Fig 1:**
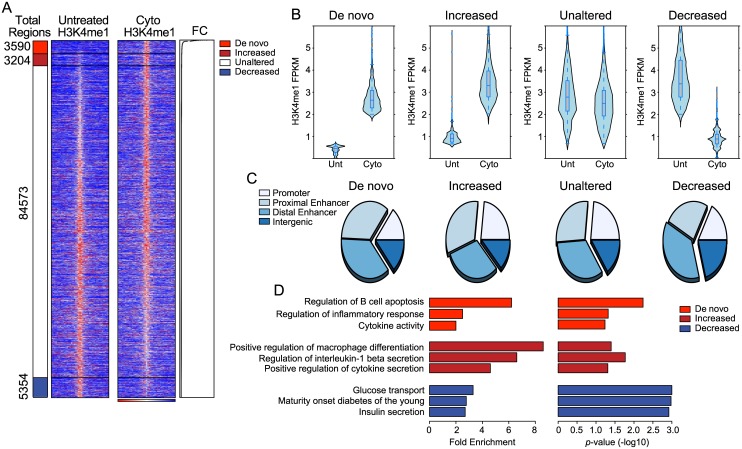
Exposure of islets to IFNγ, Il-1β, and TNFα induces changes in H3K4me1 enrichment at cis-regulatory loci and induces *de novo* enhancer formation. (A) Heatmaps of H3K4me1 read density in ±10Kb regions centered ChromHMM identified H3K4me1 enriched loci using ChIP-seq data from untreated and IFNγ, Il-1β, and TNFα (Cyto) treated islets. H3K4me1 read density is represented by the intensity of blue and red in the heatmaps: dark red indicates high read density while dark blue indicates low read density. The segregation of the regions into *de novo* regions, increased regions, unaltered regions, and decreased regions is shown on the left of the heatmaps, while the fold change (FC) of the H3K4me1 enrichment is shown on the right of the heatmaps. (B) Violin plots of the number of H3K4me1 reads (FPKM) associated with the identified H3K4me1 enriched loci in each class (*de novo*, increased, unaltered, decreased) in untreated (Unt) and IFNγ, Il-1β, and TNFα (Cyto) treated islets. (C) Distribution of the identified H3K4me1 enriched loci in each class (*de novo*, increased, unaltered, decreased) into different genomic features (promoters, proximal enhancers, distal enhancers, and intergenic). (D) Enrichment level (left graph) and p-values (right graph) of gene ontology (GO) terms enriched using genes associated with the identified *de novo*, increased, and decreased loci.

Histone 3 lysine 4 monomethylation (H3K4me1) demarcates chromatin that is in an active or poised state [24,30–33], therefore to better understand how cytokine exposure alters the transcriptional networks active in β-cells we performed ChIP-seq to identify H3K4me1 enriched regions in islets exposed to IFNγ, Il-1β, and TNFα. To better mimic the early events in T1D pathogenesis we used a relatively low dose of these cytokines (1.6ng/ml IFNγ, 0.25ng/ml Il-1β and 0.16ng/ml TNFα, or roughly 1/64th the dose typically used to induce β-cell dysfunction and apoptosis in culture [5,34]) for four days. Using these data, we identified a total of 96,721 H3K4me1 enriched regions in untreated or IFNγ, Il-1β, and TNFα treated islets (Fig 1A). Of these, 87% (84,573) had a similar level of H3K4me1 enrichment in untreated and cytokine treated islets (unaltered), 5.5% (5,354) had a reduced level of H3K4me1 enrichment in cytokine treated islets (decreased), and 3.3% (3,204) had increased H3K4me1 enrichment in cytokine treated islets (increased). In addition, we identified a further 3,590 regions significantly enriched for H3K4me1 in cytokine treated islets but that had little or no H3K4me1 enrichment in untreated islets, indicating that these regions represent *de novo* or latent cis-regulatory regions [35,36] induced by islet cytokine exposure.

In order to validate that our classification of regions into *de novo*, increased, unaltered, and decreased regions accurately reflected changes in numbers of H3K4me1 mapped reads we compared the distribution of H3K4me1 reads in each of these classes. As expected, *de novo* regions showed the largest relative increase in associated H3K4me1 reads in the cytokine treated versus the untreated samples (Fig 1B), meanwhile regions that we identified as having increased enrichment showed increased numbers of H3K4me1 reads, regions we identified as having no change in H3K4me1 enrichment had similar H3K4me1 mapped reads, and regions we identified as having reduced H3K4me1 enrichment had fewer mapped reads (Fig 1B). These results indicate that our enrichment classification scheme accurately depicts changes in H3K4me1 reads in the samples.

To determine the types of cis-regulatory regions represented by these regions, and their genomic distribution we mapped them into different classes of genomic regions. As compared to the unaltered regions, the *de novo* and decreased regions were slightly more frequently identified as being distal enhancers (20Kb to 100Kb away from the nearest transcriptional start site (TSS)). Regions with increased enrichment, on the other hand, were more frequently identified as promoters (0Kb to 2Kb away from the nearest TSS) or proximal enhancers (2Kb to 20Kb away from the nearest TSS) as compared to the unaltered regions. In all cases roughly 15% of the regions were intergenic (>100Kb away from the nearest TSS) and could not be associated with any known gene (Fig 1C). These data suggest that *de novo* and decreased regions tend to be enhancer elements, perhaps because enhancers are commonly turned on and off to fine tune gene expression.

To determine the types of genes associated with these regions we mapped them to the nearest gene and performed gene ontology (GO) enrichment analysis on the associated genes. Genes with associated *de novo* regions were specifically enriched for the GO term “Regulation of B-cell apoptosis”, although only five genes were represented in this category including *c-Myc* and *Bcl10*. Genes with associated *de novo* and increased regions were enriched for GO terms related to inflammation and immune cell activation (Fig 1D), such as “regulation of inflammatory response”, “cytokine activity” and “positive regulation of cytokine secretion”. Genes with associated decreased regions were enriched for GO terms related to β-cell function (Fig 1D), such as “glucose transport”, “Maturity onset diabetes of the young” and “insulin secretion”. These data suggest that exposure of islets to IFNγ, Il-1β, and TNFα induces the *de novo* generation, and the activation, of cis-regulatory regions associated with genes involved in the inflammatory response and in cytokine production, as well as the decreased activity of cis-regulatory regions associated with genes involved in β-cell function.

### *De novo* and increased regions are frequently enriched for H3K27me3 in untreated islets

**Fig 2:**
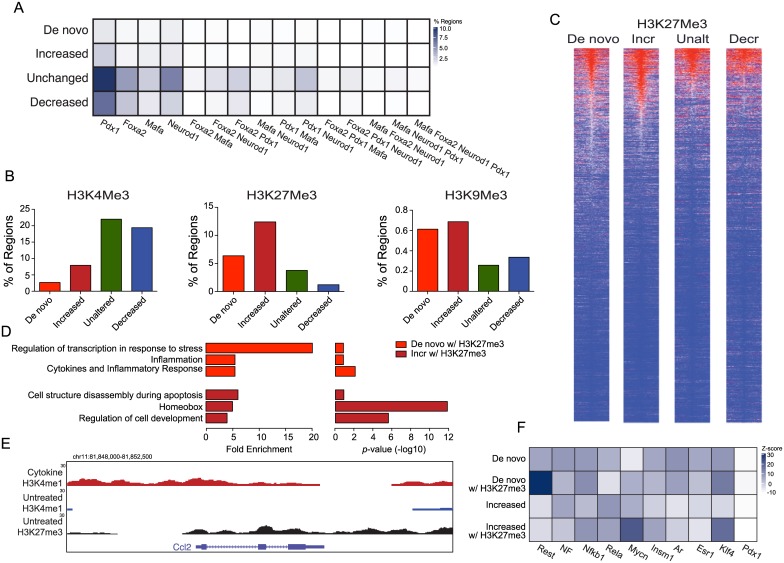
IFNγ, Il-1β, and TNFα-induced *de novo* and increased loci are frequently H3K27me3 enriched prior to cytokine exposure. (A) Heatmap of the fraction of *de novo*, increased, unaltered, and decreased loci bound by the indicated transcription factor combinations. (B) Bar graph of the fraction of *de novo*, increased, unaltered, and decreased loci pre-marked by H3K4me3 (left graph), H3K27me3 (centre graph), and H3K9me3 (right graph). (C) Heatmaps of H3K27me3 read density in ±10-kb regions centred on the identified *de novo*, increased (Incr), unaltered (Unalt), and decreased (Decr) loci in untreated islets. H3K27me3 read density is represented by the intensity of blue and red in the heatmaps: dark red indicates high read density while dark blue indicates low read density. (D) Enrichment level (left graph) and p-values (right graph) of gene ontology (GO) terms enriched using genes associated with the identified *de novo* and increased loci identified as enriched for H3K27me3 in untreated islets. (E) UCSC genome browser view of the genomic region around *Ccl2*. H3K4me1 enrichment data from IFNγ, Il-1β, and TNFα treated islets is shown in red, from untreated islets in blue, while H3K27me3 data from untreated islets is shown in black. All tracks are set to show a coverage depth range of 0 to 30. (F) Heatmap of the fraction of *de novo*, increased, unaltered, and decreased loci containing sequences that match the indicated binding motifs.

To better understand the transcriptional and epigenomic networks involved in the maintenance and function of the different classes of enhancers identified, we assessed their enrichment for Pdx1, Foxa2, Neurod1 and Mafa binding, as well as H3K4 trimethylation (H3K4me3) that is frequently enriched at active promoter regions [10,24,32,33], H3 lysine 27 trimethylation (H3K27me3) that is frequently enriched at repressed enhancer or promoter regions [10,24,33], and H3 lysine 9 trimethylation (H3K9me3) that is frequently enriched at regions within heterochromatin [10,24,33], in untreated islets. Regions that had unchanged or decreased H3K4me1 in response to cytokine treatment were more frequently occupied by Pdx1, Foxa2, Neurod1 and Mafa, and were more frequently enriched for H3K4me3 than *de novo* regions or regions that had increased H3K4me1 in response to cytokine treatment (Fig 2A and 2B). On the other hand, *de novo* regions and regions that had increased H3K4me1 in response to cytokine treatment were more frequently enriched for the repressive mark H3K27me3 (Fig 2A–2C). Very few (0.2–0.6%) of the regions in any of the classes were enriched for H3K9me3.

To determine whether the *de novo* and increased regions that were enriched for the repressive H3K27me3 mark, suggesting they were in a repressed or bivalent state respectively in untreated islets, were associated with any specific types of genes we performed gene ontology (GO) analysis on the associated genes. In fact, *de novo* regions premarked by H3K27me3 were associated with genes highly enriched for GO terms related to inflammation and the production of cytokines, while increased regions premarked with H3K27me3 were associated with genes enriched for GO terms related to factors involved in apoptosis or developmental processes (Fig 2D). These results suggest that cytokine induced inflammatory signaling in β-cells induces the generation of *de novo* enhancers at H3K27me3 repressed cis-regulatory regions associated with chemokines, such as *Ccl2* (Fig 2E), and other inflammation induced genes.

As the *de novo* and increased regions were relatively deprived in Pdx1, Foxa2, Neurod1 and Mafa binding we reasoned that they might contain binding sites for other transcription factors. In fact, the *de novo* sites premarked by H3K27me3 were particularly enriched in binding sites for Rest, a transcription factor involved in silencing neuronal genes in non-neuronal tissues [37,38] (Fig 2F). Binding sites for Nf-κB and Myc transcription factors were well enriched in the *de novo* and increased regions (Fig 2F).

### IFNγ, Il-1β, and TNFα-induced changes in H3K4me1 enrichment are associated with changes in islet transcription

**Fig 3:**
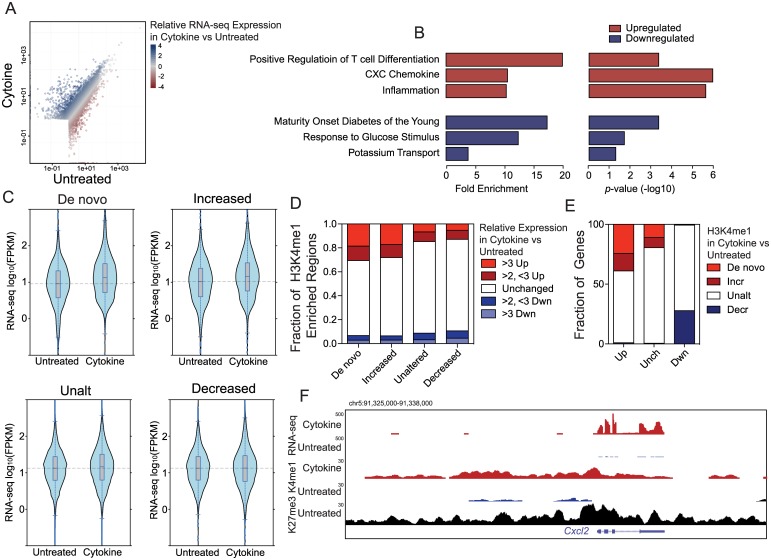
IFNγ, Il-1β, and TNFα-induced changes in H3K4me1 enrichment correspond to changes in islet transcription. (A) Scatter plot of the FPKM of identified transcripts in untreated or IFNγ, Il-1β, and TNFα (Cytokine) treated islets. The relative expression of transcripts in the IFNγ, Il-1β, and TNFα (Cytokine) treated islets as compared to in untreated islets is indicated by the colour of the dot, with darker blue indicating higher expression in IFNγ, Il-1β, and TNFα treated islets, and darker red indicating higher expression in untreated islets. Only transcripts with an FPKM of >2 in one of the samples are shown. (B) Enrichment level (left graph) and p-values (right graph) of gene ontology (GO) terms enriched using genes identified as upregulated or down regulated by IFNγ, Il-1β, and TNFα exposure. (C) Violin plots of the expression level (FPKM) of transcripts in untreated (Unt) and IFNγ, Il-1β, and TNFα (Cytokine) treated islets associated with H3K4me1 enriched loci in each class (*de novo*, increased, unaltered, decreased). (D) Bar graphs of the fraction of H3K4me1 enriched loci in each class (*de novo*, increased, unaltered, decreased) associated with transcripts up or down regulated as indicated by IFNγ, Il-1β, and TNFα exposure. (E) Bar graphs of the fraction of transcripts up or down regulated by IFNγ, Il-1β, and TNFα exposure with associated *de novo*, increased (Incr), unaltered (Unalt), decreased (Decr) loci. (F) UCSC genome browser view of the genomic region around *Cxcl2*. RNA-seq reads and H3K4me1 enrichment data from IFNγ, Il-1β, and TNFα treated islets is shown in red, from untreated islets in blue, while H3K27me3 data from untreated islets is shown in black. ChIP-seq tracks are set to show a coverage depth range of 0 to 30, RNA-seq tracks from 0 to 500.

To determine whether the identified IFNγ, Il-1β, and TNFα-induced changes in islet chromatin state corresponded with changes in transcription we performed RNA-seq on untreated and IFNγ, Il-1β, and TNFα treated islets (Fig 3A). Genes up-regulated by IFNγ, Il-1β, and TNFα exposure were enriched in GO terms related to inflammation and immune cell activation/differentiation, similar to the terms enriched using genes associated with *de novo* regions or regions with increased H3K4me1 (Fig 3B). Meanwhile, down-regulated genes were enriched for terms associated with β-cell function, again similar to what was found for genes associated with regions with decreased H3K4me1 (Fig 3B). Assessing the expression level of genes associated with the different H3K4me1-based region types identified above in untreated and IFNγ, Il-1β, and TNFα treated islets confirmed that genes associated with *de novo* and increased regions had relatively higher expression in IFNγ, Il-1β, and TNFα treated islets, as compared to untreated islets (Fig 3C). Genes associated with unchanged regions had no change in expression, and genes associated with decreased regions had lower expression in IFNγ, Il-1β, and TNFα treated islets as compared to genes associated with unchanged regions (Fig 3C). Further, *de novo* and increased regions were far more likely to be associated with genes with 2-fold or greater increase in IFNγ, Il-1β, and TNFα-induced expression, while decreased regions were more likely to be associated with genes with 2-fold or greater decrease in IFNγ, Il-1β, and TNFα-induced expression (Fig 3D). Likewise, 40% of genes up-regulated by IFNγ, Il-1β, and TNFα were associated with a *de novo* or increased region (Fig 3E) and 30% of genes down-regulated by IFNγ, Il-1β, and TNFα were associated with a decreased region (Fig 3E). The remaining up- and down-regulated genes were likely associated with modest H3K4me1 changes that didn't meet our stringent thresholds, or with other chromatin state changes, such as changes in histone acetylation. In any case, we were still able to identify 205 genes that showed an IFNγ, Il-1β, and TNFα-induced chromatin state change (from repressed or inactive to H3K4me1 enriched, i.e. the *de novo* regions), or increased H3K4me1 enrichment, that were associated with genes with up-regulated expression in response to IFNγ, Il-1β, and TNFα, and 111 genes with reduced H3K4me1 and down-regulated expression in response to IFNγ, Il-1β, and TNFα. For example, IFNγ, Il-1β, and TNFα exposure induced the transition of the cis-regulatory regions adjacent to *Cxcl2* (Fig 3F) to transition from a repressed H3K27me3 enriched state to an active H3K4me1 enriched state, and induced a dramatic 73-fold increase in its expression. These data suggest that H3K4me1 enrichment changes in islet cells are a critical part of their transcriptional response to IFNγ, Il-1β, and TNFα.

### IFNγ, Il-1β, and TNFα exposure induces *de novo* enhancer formation and cytokine gene expression in nge of 0 to 30,three hours

**Fig 4:**
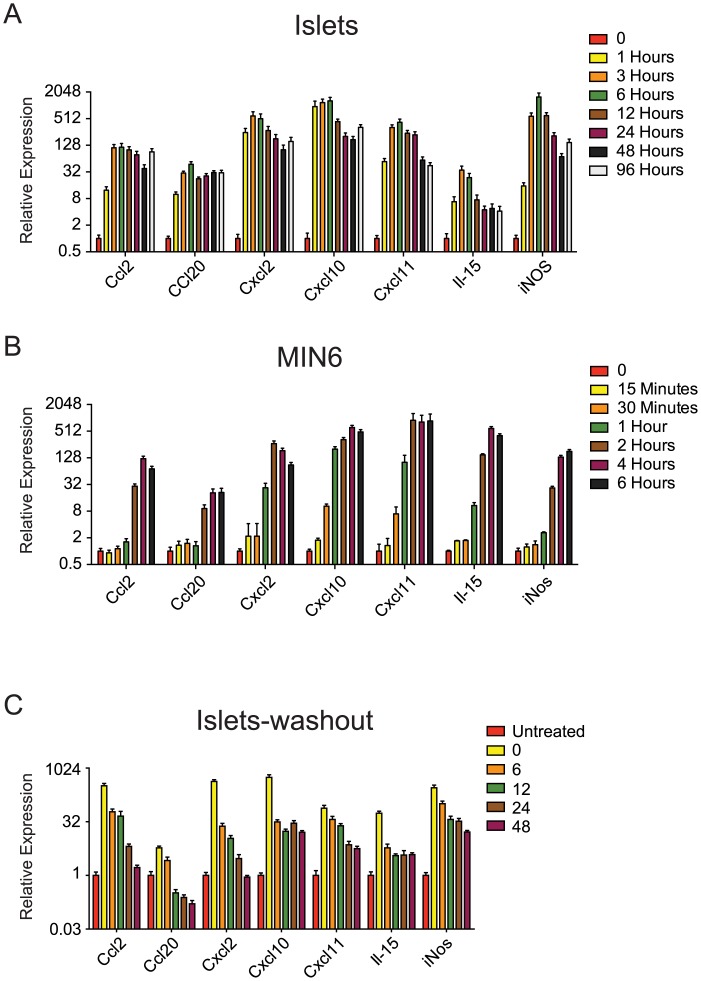
IFNγ, Il-1β, and TNFα induce cytokine gene expression within three hours in β-cells. (A) Time course of the relative expression of the indicated chemokines and cytokines in islets exposed to IFNγ, Il-1β, and TNFα as determined by qPCR. (B) Time course of the relative expression of the indicated chemokines and cytokines in MIN6 cells exposed to IFNγ, Il-1β, and TNFα as determined by qPCR. (C) Time course of the relative expression of the indicated chemokines and cytokines in islets exposed to IFNγ, Il-1β, and TNFα for three hours then placed in media without IFNγ, Il-1β, and TNFα for the indicated time points as determined by qPCR.

Our data above suggested that many chemokines and cytokines expressed by β-cells in response to cytokine exposure had associated *de novo* or increased regions that were H3K27me3 enriched in untreated cells, including *Ccl2*, *Ccl20*, *Cxcl2*, *Cxcl10*, *Cxcl11*, *Il-15*, and others. This suggests that these cis-regulatory regions are normally in a repressed (H3K27me3 enriched) or bivalent (H3K4me1 and H3K27me3 enriched) state, and undergo a chromatin state transition to an active state upon cytokine exposure, with concomitant increased gene expression. To determine the time frame in which these chromatin state and gene expression alterations might take place we first performed a gene expression time course in isolated islets treated with IFNγ, Il-1β, and TNFα (Fig 4A). Even after only one hour significant increases in all the genes assessed were noted, with maximal expression reached within 3–6 hours (Fig 4A), reaching between 30- and 1,200-fold increases in expression as compared to time 0. Thereafter, expression was either maintained or marginally decreased over the following four day time course. To ensure these expression changes were actually occurring in β-cells, and not in contaminating immune cells within the islets, we performed a similar time course using MIN6 cells, a common β-cell line. As was found with islets, peak induction of the genes was reached at between two and four hours (Fig 4B). Similar levels of induction (Fig 4B) between the islets and MIN6 cells were obtained, indicating the gene expression changes noted in islets are largely the result of changes in gene expression in β-cells. We next asked how long the expression of these genes persists after removal of IFNγ, Il-1β, and TNFα. In fact, we found that even 48hrs after IFNγ, Il-1β, and TNFα removal the expression of *Ccl2*, *Cxcl10*, and *Cxcl11* are still significantly elevated as compared to untreated cells (5.9 fold, 33.25 fold, 36.6 fold respectively, p<0.05) (Fig 4C).

**Fig 5:**
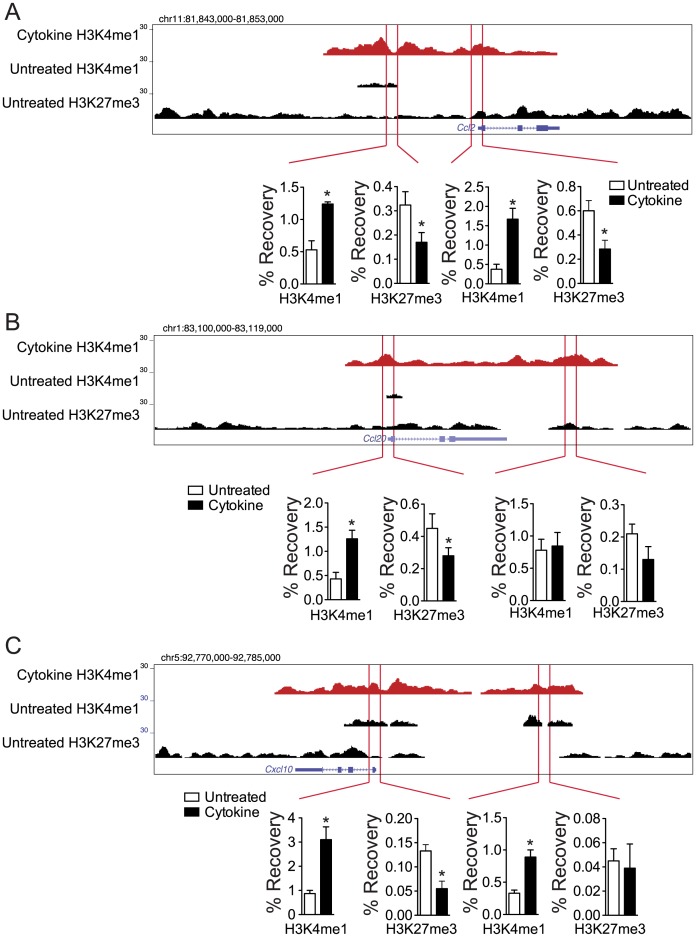
IFNγ, Il-1β, and TNFα induce chromatin state changes at promoters and enhancers for chemokine genes within three hours in β-cells. Enrichment of H3K4me1 and H3K27me3 (% Recovery) at promoter and enhancer regions indicated by the red boundaries, around (A) *Ccl2*, (B) *Ccl20*, and (C) *Cxcl10* in MIN6 cells untreated or treated with IFNγ, Il-1β, and TNFα for three hours.

To confirm that the induction of these genes by IFNγ, Il-1β, and TNFα is associated with corresponding chromatin state changes we performed ChIP-qPCR to assess H3K4me1 and H3K27me3 enrichment in MIN6 cells untreated or IFNγ, Il-1β, and TNFα treated for three hours. All of the selected loci adjacent to *Ccl2* (Fig 5A), *Ccl20* (Fig 5B) and *Cxcl10* (Fig 5C), but one, showed increased H3K4me1 enrichment and corresponding H3K27me3 reductions after only three hours of exposure to IFNγ, Il-1β, and TNFα. Taken together, these results indicate that IFNγ, Il-1β, and TNFα can induce the activation of otherwise repressed cis-regulatory loci and thereby significantly up-regulate the expression of various chemokine and cytokine genes in β-cells in as a little as three hours, and that these changes likely persist for hours to days after cytokine stimulation is removed.

### Trithorax group complex proteins are expressed in essed i

**Fig 6:**
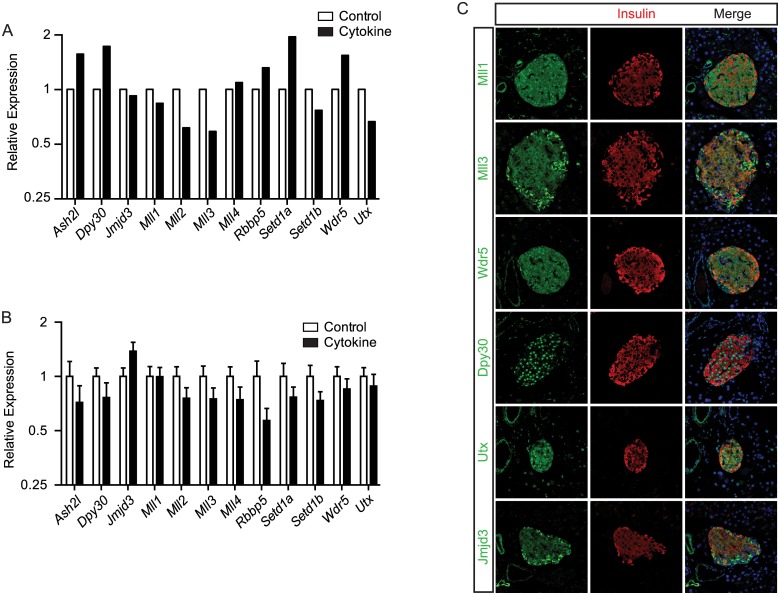
TrxG complex proteins are expressed in adult islet cells and are largely non-responsive to IFNγ, Il-1β, and TNFα exposure. (A) Expression level (FPKM) of the genes for the primary proteins in the TrxG complexes in untreated and IFNγ, Il-1β, and TNFα (cytokine) treated islets. (B) qPCR validation of the unaltered expression of the genes for the primary proteins in the TrxG complexes in IFNγ, Il-1β, and TNFα (cytokine) treated islets as compared to untreated islets. (C) Immunohistochemical co-staining of selected TrxG proteins (green) and insulin (red) in adult pancreas sections. Topro staining (blue) is shown in the merged image to identify nuclei.

The monomethylation of H3K4 is carried out by SET domain containing histone methyltransferases; specifically Mll3 is thought to largely be responsible for *de novo* enhancer formation in mammalian cells [39,40]. Meanwhile, H3K27me3 demethylation is carried out by Jmjd3 or Utx [41,42]. All of these proteins are thought to be associated with trithorax group (TrxG) protein complexes [13,17,43]. These TrxG complexes can be classified based on which SET domain containing histone methyltransferase protein they contain (Setd1a, Setd1b, Mll1, Mll2, Mll3, or Mll4), but all of the complex variants contain the core proteins Ash2l, Dpy30, Wdr5, and Rbbp5 [40,44]. To confirm the expression of these TrxG complex proteins in β-cells and to determine whether their expression is altered by IFNγ, Il-1β, and TNFα we first assessed their expression in our RNA-seq libraries. These data indicate that all of these proteins are present in islets, although, Mll2 and Set1b are present at relatively lower levels (Fig 6A). Further they indicate that the expression of none of these genes is significantly altered by IFNγ, Il-1β, and TNFα, either in islets (Fig 6A) or in MIN6 cells exposed to these cytokines for three hours (Fig 6B). Finally to confirm the expression of these factors in β-cells *in vivo* we performed immunohistochemistry on adult pancreas sections. These results showed that Mll1, Mll3, Ash2l, Wdr5, Rbbp5, Jmjd3, and Utx, are all expressed in insulin positive cells (Fig 6C). Interestingly, although Dpy30 staining was predominantly nuclear, all the other proteins showed staining in both the cytoplasm and the nucleus as has been previously determined [45–48]. Together, these data confirm that TrxG proteins are present within β-cells and, given that these complexes can contain both H3K4 histone methyltransferase activity and H3K27me3 demethylase activity, are likely at least partly responsible for the IFNγ, Il-1β, and TNFα mediated conversion of inactive, repressed and bivalent cis-regulatory loci into active loci.

### Suppression of the core TrxG complex protein *Wdr5* inhibits IFNγ, Il-1β, and TNFα-induced chemokine and cytokine gene expression in in *Wdr5*

**Fig 7:**
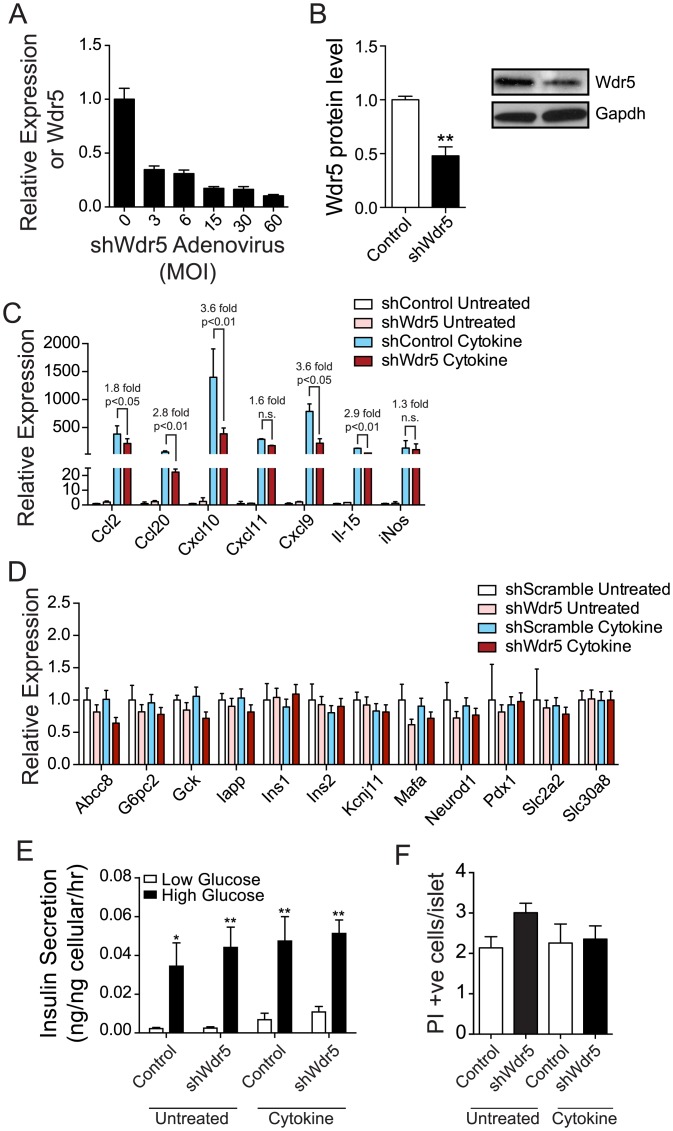
Suppression of *Wdr5*, a core component of the TrxG complexes, inhibits IFNγ, Il-1β, and TNFα-induced chemokine gene expression in islets without affecting β-cell function. (A) *Wdr5* expression in response to a titration of pAd-shWdr5 in islets as determined by qPCR. (B) Western blot analysis of Wdr5 protein levels in islets transduced with an sh*Wdr5* or control adenovirus, Gapdh protein levels were used to normalize the protein loading between the samples. Representative Wdr5 and matching Gapdh blots are shown. (C) Expression of selected chemokine and cytokine genes in islets transduced with shScramble or sh*Wdr5*-expressing adenoviruses then untreated or treated with IFNγ, Il-1β, and TNFα (cytokine) as determined by qPCR. (D) Expression of selected genes involved in regulating β-cell function in islets transduced with shScramble or sh*Wdr5*-expressing adenoviruses then untreated or treated with IFNγ, Il-1β, and TNFα (cytokine) as determined by qPCR. (E) Glucose-induced insulin secretion assays of islets transduced with shScramble or sh*Wdr5*-expressing adenoviruses then untreated or treated with IFNγ, Il-1β, and TNFα (cytokine). (F) Propidium iodide (PI) incorporation in islets transduced with shScramble or sh*Wdr5*-expressing adenoviruses then untreated or treated with IFNγ, Il-1β, and TNFα (cytokine).

The core TrxG protein Wdr5 is essential for the function of the TrxG complex [43,49–51]. Based on our data above we hypothesized *Wdr5* suppression, and thus TrxG complex disruption, would inhibit IFNγ, Il-1β, and TNFα-induced activation of inactive, repressed, and bivalent cis-regulatory loci. As such, we developed an adenovirus expressing shRNA’s targeting *Wdr5*. Transduction of dispersed islet cells with this adenovirus resulted in ~85% suppression of *Wdr5* transcript at an MOI of 15 (Fig 7A), although this resulted in only a 52% (p<0.01) reduction in Wdr5 protein after 48 hours (Fig 7B). Despite this, suppression of *Wdr5* in dispersed islets for 48 hours did result in significant reductions in the induction of *Ccl2* (1.8 fold, p<0.05), *Ccl20* (2.8 fold, p<0.01), *Cxcl10* (3.6 fold, p<0.01), *Cxcl9* (3.6 fold, p<0.05), and *Il-15* (2.9 fold, p<0.01) (Fig 7B) by exposure to IFNγ, Il-1β, and TNFα for three hours. To ensure this reduction in IFNγ, Il-1β, and TNFα-induced chemokine expression didn't occur at the expense of β-cell function or survival we next assessed the expression of key markers of β-cell function, including *Abcc8*, *G6pc2*, *Gck*, *Iapp*, *Ins1*, *Ins2*, *Kcnj11*, *Mafa*, *Neurod1*, *Pdx1*, *Slc2a2*, and *Slc30a8* [52–57]. Neither *Wdr5* suppression for 48 hours nor three hours of exposure to IFNγ, Il-1β, and TNFα, at the dose used, had any significant effect on the expression of any of the twelve β-cell genes tested (Fig 7C). Nor did we find that these treatments had any effect on glucose-stimulated insulin secretion (Fig 7D), or apoptosis (Fig 7E).

The expression of *Ccl2*, *Ccl20*, *Cxcl10*, *Cxcl11*, *Cxcl9*, *iNos*, or *Il-15* were still relatively highly expressed in response to IFNγ, Il-1β, and TNFα, even after *Wdr5* suppression, likely in part due to the high level of Wdr5 protein remaining. To determine whether longer periods of *Wdr5* suppression would results in greater levels of Wdr5 protein reduction, and thus greater levels of chemokine inhibition, we cultured transduced dispersed islet cells for four days. After this time Wdr5 protein levels were similarly reduced (46%, p<0.05, S1 Fig) and this did not substantially decrease IFNγ, Il-1β, and TNFα induced *Ccl2*, *Ccl20*, *Cxcl10*, *Cxcl11*, *Cxcl9*, *iNos*, or *Il-15* expression beyond what we found after 48hrs of *Wdr5* suppression. However, *Abcc8*, *G6pc2*, *Ins2*, *Mafa*, *Slc2a2*, and *Slc30a8* became marginally down-regulated (S1 Fig). As we found at 48 hours suppression of *Wdr5* did not have any effect on glucose-stimulated insulin secretion, or apoptosis (S1 Fig); although, the culture period itself did reduce the level of insulin secreted in response to high glucose in both the control and *Wdr5* shRNA treated samples.

### Inhibition of Jmjd3/Utx by the small molecule GSK-J4 significantly inhibits IFNγ, Il-1β, and TNFα-induced chemokine and cytokine gene expression in y inhib

**Fig 8:**
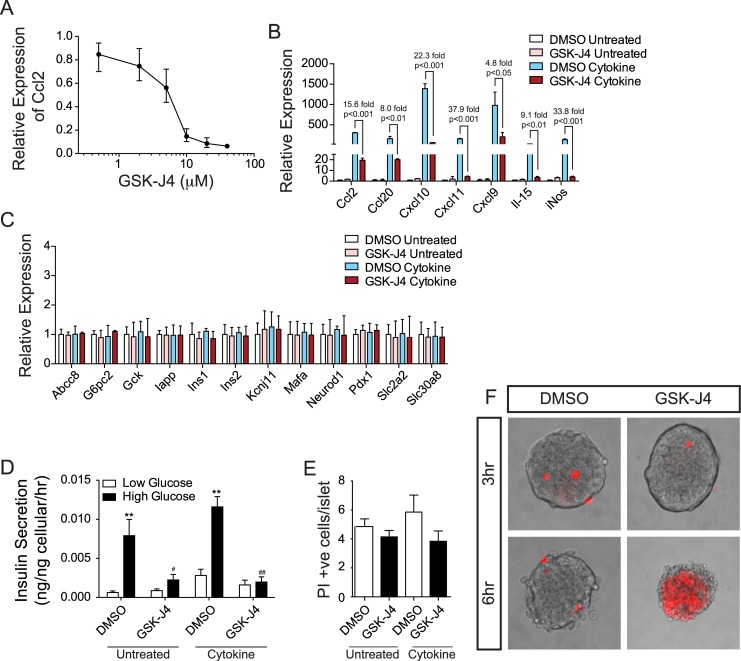
The small molecule GSK-J4, an inhibitor of Jmjd3 and Utx, significantly blunts IFNγ, Il-1β, and TNFα-induced chemokine gene expression in islets. (A) A dose-response curve of *Ccl2* expression in islets exposed a titration of concentrations of GSK-J4 as determined by qPCR. (B) Expression of selected chemokine and cytokine genes in islets exposed to 20μM GSK-J4 or a vehicle control (DMSO) and simultaneously untreated or treated with IFNγ, Il-1β, and TNFα (cytokine) as determined by qPCR. (C) Expression of selected genes involved in regulating β-cell function in islets exposed to 20μM GSK-J4 or a vehicle control (DMSO) and simultaneously untreated or treated with IFNγ, Il-1β, and TNFα (cytokine) as determined by qPCR. (D) Glucose-induced insulin secretion assays of islets exposed to 20μM GSK-J4 or a vehicle control (DMSO) and simultaneously untreated or treated with IFNγ, Il-1β, and TNFα (cytokine) as determined by qPCR. (E) Propidium iodide (PI) incorporation in islets exposed to 20μM GSK-J4 or a vehicle control (DMSO) and simultaneously untreated or treated with IFNγ, Il-1β, and TNFα (cytokine) as determined by qPCR. (F) Representative images of islets treated with DMSO or GSK for three and six hours respectively. PI positive cells are marked in red.

Our data above indicated that targeting the TrxG complex to prevent IFNγ, Il-1β, and TNFα-induced chromatin state changes may be a promising approach to prevent cytokine induced chemokine and cytokine gene expression in β-cells. To seek a more effective approach we next used the ethyl ester pro-drug GSK-J4 that is converted to GSK-J1, a selective inhibitor of Jmjd3 and Utx [58,59], within the cells. We hypothesized this approach might be effective as we had determined that many of the cis-regulatory loci adjacent to the cytokine induced chemokine and cytokine genes are normally in a H3K27me3 enriched, repressive or bivalent state, and thus require H3K27me3 removal for activation. We first performed a dose-response curve and found that 20μM GSK-J4 resulted in greater than 90% suppression of IFNγ, Il-1β, and TNFα-induced *Ccl2* gene expression in islets (Fig 8A). Using this dose on islets treated with IFNγ, Il-1β, and TNFα for three hours resulted in highly significant reductions in the induction of *Ccl2* (15.6 fold, p<0.001), *Ccl20* (8.0 fold, p<0.01), *Cxcl10* (22.3 fold, p<0.001), *Cxcl11* (37.9 fold, p<0.001), *Cxcl9* (4.8 fold, p<0.05), *Il-15* (9.1 fold, p<0.01), and in *iNos* (33.8 fold, p<0.001) (Fig 8B). Further, exposure of islets to this dose of GSK-J4 for three hours, in the presence or absence of IFNγ, Il-1β, and TNFα had no significant effect on the expression of genes involved in β-cell function (Fig 8C). However, it did significantly decrease glucose-stimulated insulin secretion (Fig 8D). Also, although GSK-J4 did not increase islet cell apoptosis after three hours (Fig 8E and 8F), after six hours of exposure to GSK-J4 almost all the islet-cells were PI positive suggesting they had undergone apoptosis (Fig 8F). Lower concentrations of GSK-J4 (down to 2μM) also resulted in significant cell death after six hours (S2 Fig) indicating the use of lower doses of GSK-J4 for longer treatment periods is not a viable approach to reduce IFNγ, Il-1β, and TNFα-induced chemokine gene expression in islets. Despite this, these results further confirm that targeting the TrxG complex may be a useful approach to prevent cytokine induced chemokine and cytokine gene expression in β-cells; however, alternative mechanisms need to be sought.

## Discussion

H3K4me1, H3K4me2, H3K4me3, H3K9ac and H3K27ac have proven to be particularly useful histone modifications for the identification of promoters and enhancers, and for their classification into regions that are in an active or poised (ready to be active) state [24,31–33]. In previous work, we found H3K4me1 to be particularly useful for the identification of such elements in pancreatic islets [30], as it is enriched at both enhancers and promoters in an active or poised state [24,30–33]. H3K4me2, on the other hand, typically co-occurs with H3K4me1 and this mark is therefore largely redundant to H3K4me1 for the identification of promoters and enhancers, H3K4me3 is only enriched at promoters and strong enhancers, and is thus generally not useful for the identification of enhancers, and H3K9ac and H3K27ac are enriched only at active, but not poised, promoters and enhancers. The downfall of using H3K4me1 enrichment data alone to identify promoter and enhancer loci is that it cannot be used to discriminate between active and poised loci, for this H3K9ac and/or H3K27ac data is necessary. The activation of poised loci is thought, however, to correlate with increases in H3K4me1 enrichment, which may be the case for many of our identified H3K4me1-increased loci.

H3K4me1 or H3K4me2, can, unlike H3K9ac and/or H3K27ac, however, be used to identify cis-regulatory loci generated *de novo* (i.e. loci induced to convert from a repressed or unmarked state to an active or poised state) [35,36]. The ability of mature cells to generate *de novo* enhancers in response to specific environmental cues has only recently been described [35,36], and never previously shown in β-cells. Using our H3K4me1 data we were able to identify 3,520 such loci specifically induced by exposure to IFNγ, Il-1β, and TNFα. Work done in macrophages suggests that the majority of *de novo*, or latent, enhancers are unique to specific stimuli [35]. Thus, the ability of other factors important in diabetes pathology, such as gluco- and lipotoxicity, ER stress etc., to induce *de novo* enhancer generation in β-cells, and the full complement of possible *de novo* enhancers that can be generated in β-cells, remains to be determined.

Of interest, we show that many IFNγ, Il-1β, and TNFα-induced chemokines and cytokines in β-cells, including *Ccl2* (*Mcp-1*), *Ccl20* (*Mip-3α*), *Cxcl9*, *Cxcl10* (*Ip-10*), *Cxcl11*, and *Il-15* are associated with *de novo* or H3K4me1 increased promoters and enhancers that are enriched for H3K27me3 in untreated cells. H3K27me3 is associated with polycomb complex group (PcG) recruitment [12,13], and it is normally found at repressed (H3K27me3 enriched) and bivalent (H3K4 methylation and H3K27me3 enriched) cis-regulatory loci [60]. The enrichment of H3K27me3 at the promoters and enhancers of these chemokine and cytokine genes in untreated islets suggests they require active repression to prevent their promiscuous activity, and that this repression needs to be overcome to enable their expression.

Many of the IFNγ, Il-1β, and TNFα-induced de novo and increased regions were not associated with genes up-regulated by IFNγ, Il-1β, and TNFα exposure. Likewise, many of the IFNγ, Il-1β, and TNFα-induced decreased regions were not associated with genes down-regulated by IFNγ, Il-1β, and TNFα. This seemingly low level of correspondence between these data sets is, however, unsurprising. This is due in part to the complexity of transcriptional regulation, with the vast majority of genes having several associated enhancers, not all of which have the same increases and decreases in H3K4me1, meaning that changes in H3K4me1 at one enhancer may have little overall impact on the gene’s total expression, or may in fact be nullified by opposing changes in H3K4me1 at another locus. Additionally, many of the induced changes in H3K4me1 enrichment or gene expression may not have been sufficient to meet the stringent thresholds used. Further, the heterogeneity in the cell types present in islets, which were used to generate these data, adds to the complexity. Specifically it may be the case that relatively few cells can up-regulate a transcript sufficiently to be picked up using our RNA-seq data, but the contribution these few cells make to the ChIP-seq data may not be sufficient for any H3K4me1 enrichment signal at the promoter or enhancers associated with the gene to show up. Also, in many cases the association of a H3K4me1 region to the nearest gene TSS may not be correct, although, it is thought this method of enhancer-gene association is correct the majority of the time (>66%) [26]. Regardless of these issues, it is clear that IFNγ, Il-1β, and TNFα-induced H3K4me1 increases were typically associated with increased transcription of associated genes (Fig 3C).

In order to best mimic the early stages of T1D pathogenesis *in vitro* for our ChIP-seq and RNA-seq experiments we used a relatively low dose of IFNγ, Il-1β, and TNFα for a longer time frame than normally used (~1/64th the commonly used dosage for four days [5,34]). Despite this, we were surprised to find maximal chemokine and cytokine gene expression was achieved after only 3–6 hours of IFNγ, Il-1β, and TNFα exposure. Likely this is true for many of the IFNγ, Il-1β, and TNFα up-regulated transcripts identified in our RNA-seq data. We confirmed that even after only three hours IFNγ, Il-1β, and TNFα induced increases in H3K4me1, and decreases in H3K27me3, at promoters and enhancers associated with IFNγ, Il-1β, and TNFα-induced chemokine and cytokine genes. Further, our data shows that once these enhancers are formed they subsequently continue to drive gene expression for hours to days, even in the absence of continued IFNγ, Il-1β, and TNFα exposure. These results suggest that even a short period of exposure to immune cell produced proinflammatory cytokines can have a profound influence on β-cell production of factors that help drive continued inflammatory processes.

H3K4 methylation is catalyzed by Set/MLL histone methyltransferase family proteins including Set1a, Set1b, Mll1, Mll2, Mll3, and Mll4, as well as the SET domain containing proteins Setd7 and Smyd1-6 [61,62]. H3K27me3 is demethylated by Utx or Jmjd3 (also known as Kdm6a and Kdm6b) [41,42]. All of these proteins, with the exceptions of Setd7 and Smyd1-6, are found within Trithorax group (trxG) protein complexes [17,40,44]. These complexes all contain the core proteins Ash2l, Dpy30, Rbbp5, and Wdr5 that are critical to their assembly and function, which are thus essential for many developmental processes [43,51,63–65]. As such, we hypothesized that the TrxG complex may be a useful target for the prevention of the cytokine-induced activation of promoters and enhancers associated with chemokine and cytokine genes in β-cells. For this we took two different approaches. Our first approach was to suppress *Wdr5*, as this protein is necessary for TrxG complex formation and activity [43,63]. Although, this did significantly reduce IFNγ, Il-1β, and TNFα-induced expression of *Ccl2*, *Ccl20*, *Cxcl10*, *Cxcl9*, and *Il-15*, these reductions were relatively modest (1.8 to 3.6 fold). This suggests that the level of *Wdr5* suppression achieved (only ~50% at the protein level) was not sufficient to completely block TrxG complex activity, or that other H3K4 methyltransferases independent of the TrxG complex, such as Setd7 [66,67], are involved. As an alternative approach we chose to use the small molecule inhibitor GSK-J4, which is converted intracellularly into GSK-J1, a potent inhibitor of Jmjd3 and Utx [58,59]. We hypothesized that as the majority of cytokine induced chemokine and cytokine genes are in an H3K27me3 repressed state in β-cells, inhibition of these H3K27me3 demethylases, would prevent their activation. In fact, treatment of cytokine exposed islets with this inhibitor lead to between 8 and 37.9 fold reductions in IFNγ, Il-1β, and TNFα-induced expression of all of the chemokines and cytokines tested. These results are in agreement with the finding that GSK-J4 can inhibit LPS induced TNFα production by macrophages [58]. Despite these promising results it should be noted that GSK-J4 exposure did induce significant β-cell dysfunction, and even at relatively low doses significant numbers of islet cells were apoptotic after six hours of exposure, suggesting that islet cells may be dependent upon p16^INK4A^ expression, which is dependent upon Jmjd3/Utx mediated repression of Cdk4/Cdk6, for their survival [68]. The fact that neither *Wdr5* suppression nor Jmjd3/Utx inhibition resulted in significant reductions in the expression of key genes involved in β-cell function, however, suggests that targeting this complex, and specifically Jmjd3 and/or Utx, may be a valuable way to prevent cytokine induced chemokine and cytokine gene expression in β-cells without compromising β-cell function. Despite this other approaches to targeting these proteins need to be sought.

## Materials and Methods

### Mouse maintenance and islet isolations

Mice were maintained according to the guidelines of the Canadian Council on Animal Care. All protocols were approved by the University of British Columbia Animal Care Committee. Islets were isolated as previously described [30,69]. Briefly, pancreata from both male and female mice were perfused with 1000U/ml Collagenase XI (Sigma-Aldrich) and incubated for 15 minutes at 37°C. Pancreata were manually disrupted and passed through a 70μM filter (Corning) and islets were hand-picked. Isolated islets were allowed to recover for 4–6 hours prior to use in experiments. Islets were cultured in RPMI 1640 supplemented with 10% FBS, 50U/ml Penicillin/Streptomycin and 2mM L-Glutamine at 37° in a 5% CO_2_ humidified incubator with or without 1.6ng/ml IFNγ, 0.25ng/ml Il-1β and 0.16ng/ml TNFα (eBioscience) as indicated.

### Chromatin immunoprecipitation sequencing (ChIP-seq) and RNA sequencing (RNA-seq)

Chromatin immunoprecipitation (ChIP) was performed using 3μg of anti-H3K4me1 (Abcam, Toronto, ON, Canada) with islets from at least 10 adult ICR mice (8–10 weeks old) exposed to 1.6ng/ml IFNγ, 0.25ng/ml Il-1β and 0.16ng/ml TNFα for four days. DNA from 4 separate ChIP experiments was pooled for library construction and sequencing as previously described [29,30]. Obtained reads from IFNγ, Il-1β and TNFα treated and untreated libraries were mapped to the NCBI37/mm9 genome using bowtie2 [70]. ChromHMM [71] was then used to identify H3K4me1 enriched regions in untreated and IFNγ, Il-1β and TNFα treated islets. These loci were merged to produce a region set of all identified H3K4me1-enriched loci. This list was then filtered to remove loci with only very low levels of H3K4me1 enrichment (FPKM<2 in both samples). Regions were considered *de novo* if they had a FPKM<0.66 in untreated islets, but FPKM>2 in the IFNγ, Il-1β and TNFα treated sample. Regions were considered increased if they had FPKM fold increase of >3 fold, and decreased if they had an FPKM decrease of >3 fold in the treated versus the untreated sample. Binding of Pdx1, Mafa, Neurod1, and Foxa2, as well as enrichment of H3K4me3, H3K27me3, and H3K9me3 at these loci was determined using previously obtained data [29,30].

For RNA-seq islets from at least four adult ICR mice were treated as described above. Total RNA from the islets was pooled and used for library generation and sequencing as previously described [30]. TopHat and Cufflinks [72] were used to map the reads to known UCSC transcripts and to identify differentially expressed genes. Only transcripts with a FPKM greater than 2 were considered expressed and used in subsequent analyses. H3K4me1 enriched loci identified as described above were associated with transcripts with the closest start site within 100Kb. Gene ontology enrichment analyses were carried out using DAVID [73]. All data were deposited under GEO accession GSE73289.

### qPCR analyses

RNA from MIN6 cells or islets treated as indicated was isolated using Trizol (Life Technologies) and purified using RNA purification columns. All primers were designed using Primer3Plus and spanned introns where possible; primer sequences are available on request. A Viia7 real-time PCR system (Applied Biosystems) and Fast SYBR Green Master Mix (Applied Biosystems) was used for all reactions. cDNAs from at least triplicate experiments were obtained by reverse transcription of 1μg total RNA. A 10ng amount of generated cDNA was used in each reaction, and all reactions were performed in triplicate. Obtained values were normalised to β-actin Ct values and the change in expression was calculated using the 2^-ΔΔCt^ method.

### Chromatin immunoprecipitation with quantitative PCR (ChIP-qPCR)

For ChIP-qPCR, we performed ChIP on ~10^6^ MIN6 cells treated or untreated with IFNγ, Il-1β and TNFα as described above for three hours using 3μg of anti-H3K4me1 (Abcam) or anti-H3K27me3 (Abcam). ChIPs were performed in quadruplicate and the obtained input and ChIP DNA analyzed using primers to the indicated promoter and enhancer regions, or to two different negative control regions, with an ABI Viia7 real-time PCR system (Applied Biosystems) and Fast SYBR Green Master Mix (Applied Biosystems). The %recovery of each target site was calculated using the cycle difference between the input and ChIP DNA. Primers were designed using Primer3 and are available upon request.

### Immunohistochemistry

Immunostaining was performed on paraformaldehyde (PFA)-fixed paraffin-embedded adult pancreas tissues as previously described [69]. Primary antibodies included rabbit anti-Mll1 (1/250; Bethyl Laboratories), rabbit anti-Mll3 (1/250; Abcam), rabbit anti-Wdr5 (1/2500; Bethyl Laboratories), rabbit anti-Dpy30 (1/500; Abcam), rabbit anti-Jmjd3 (1/50; Millipore), rabbit anti-Utx (1/2000; Millipore), and guinea pig anti-insulin (1/1000; Abcam). Fluorescently conjugated secondary antibodies included donkey anti-rabbit Alexa 488 (1/1000; Molecular Probes) and goat anti-guinea pig Alexa 546 (1/500; Molecular Probes). Sections were imaged with an SP8 confocal microscope (Leica).

### Generation of pAd-sh*Wdr5* adenovirus and utilization of GSK-J4

Three different pLKO vectors containing short hairpin constructs targeting Wdr5 under control of the hU6 promoter, and a scramble shRNA construct, were purchased from OpenBiosystems. These shWdr5 expressing vectors were prescreened for their ability to suppress Wdr5 in MIN6 cells. Subsequently, the optimal targeting sequence that suppressed Wdr5 by ~90%, and the scramble sequence, were inserted into pAdTrack using InFusion cloning (Clontech) and sequence verified. These were then used to make the pAdV-shWdr5 and pAdV-shScramble adenoviruses using the pAdEasy system as previously described [69]. Islets were transduced with these adenoviruses at the indicated MOI’s for three hours and 48 hours later were treated with or without IFNγ, Il-1β and TNFα, as indicated, for a subsequent three hours. GSK-J4 (Sigma-Aldrich) or DMSO was used to treat islets at the concentrations indicated with or without IFNγ, Il-1β and TNFα for three hours.

### Quantification of apoptosis and glucose-stimulated insulin secretion assays

To identify apoptotic cells islets, cultured and treated as described above, were incubated with 1μM propidium iodide (PI) (Sigma-Aldrich) for 30 minutes. PI positive cells were manually counted using an Olympus CKX41 inverted, fluorescent microscope and the number of PI positive cells determined for 50 islets from each of three independent experiments. For glucose-stimulated insulin secretion assays transduced and untransduced islets were washed and equilibrated in Kreb’s Ringer Buffer (KRB) (115mM NaCl, 5mM KCl, 24mM NaHCO3, 2.5mM CaCl2, 1mM MgCl2, 10mM HEPES and 2% w/v BSA) with 2.8mM glucose for three hours. Transduced and untransduced islets were treated with or without cytokine during this time while only untransduced islets were treated with or without GSK-J4. Islets were then transferred into 500μL KRB with 2.8mM glucose for 30min followed by a further 30min incubation in 16.7mM glucose. Supernatants were collected to measure insulin secretion and islets were lysed in 500μL acid ethanol (0.5ml HCl in 50ml 70% EtOH) to measure cellular insulin. All samples were analysed using the Insulin (Mouse) ELISA (Alpco) and plates were read using a Multiscan Ascent plate reader (Thermo LabSystems).

### Western blot analysis

Islets were isolated as described above and allowed to recover for 4–6 hours. Islets were dispersed and plated on 804G, a complete extracellular-matrix (ECM) produced by a rat bladder carcinoma cell line, treated tissue culture dishes at ~200 islet equivalents per well. Cells were cultured overnight and transduced with the pAdV-shWdr5 and pAdV-shScramble adenoviruses at an MOI of 10. The media was changed 24 hours later and cells were cultured for a further 24 to 72 hours before harvesting. Cells were collected by trypsinization and lysed in 50μL RIPA (Thermofisher) supplemented with 1x Halt Protease Inhibitor (Thermofisher). 25μg of total protein was loaded in each well of a 4–15% Tgx gel (Biorad). Membranes were probed with antibodies against: Wdr5 (1/500; Bethyl) and Gapdh (1/10000, Cell Signaling). Primary antibodies were detected with Donkey anti-Rabbit HRP (1/10000, Santa Cruz).

### Statistical analysis

Statistical analysis was performed using the paired, two-tailed student’s t-test, with the exception of qPCR data for which the non-parametric Mann Whitney test was used to compare the ΔCt’s of the reference and the sample using Prism6 (GraphPad Software). Data from at least three independent experiments are represented as mean +/- S.E.M. Statistical significance was accepted at P values <0.05.

## Supporting Information

S1 Fig Suppression of *Wdr5* for four days does not increase inhibition of IFNe, Il-1not increase inhibition of ry construction and sequencing.ility at the CFRI fuppression. (A) Western blot analysis of Wdr5 protein levels in islets transduced with an sh*Wdr5* or control adenovirus, Gapdh protein levels were used to normalize the protein loading between the samples. Representative Wdr5 and matching Gapdh blots are shown. (B) Expression of selected chemokine and cytokine genes in islets transduced with shScramble or sh*Wdr5*-expressing adenoviruses then untreated or treated with IFNγ, Il-1β, and TNFα (cytokine) as determined by qPCR. (C) Expression of selected genes involved in regulating β-cell function in islets transduced with shScramble or sh*Wdr5*-expressing adenoviruses then untreated or treated with IFNγ, Il-1β, and TNFα (cytokine) as determined by qPCR. (D) Glucose-induced insulin secretion assays of islets transduced with shScramble or sh*Wdr5*-expressing adenoviruses then untreated or treated with IFNγ, Il-1β, and TNFα (cytokine). (E) Propidium iodide (PI) incorporation in islets transduced with shScramble or sh*Wdr5*-expressing adenoviruses then untreated or treated with IFNγ, Il-1β, and TNFα (cytokine).
(EPS)

S2 Fig Exposure of islets to low dose GSK-J4 for six hours induces significant cell death. (A) Representative images of islets treated with DMSO or the indicated concentration of GSK for six hours. PI positive cells are marked in red.
(EPS)
